# STING-pathway modulation to enhance the immunogenicity of adenoviral-vectored vaccines

**DOI:** 10.1038/s41598-022-18750-3

**Published:** 2022-08-24

**Authors:** Eriko Padron-Regalado, Marta Ulaszewska, Alexander D. Douglas, Adrian V. S. Hill, Alexandra J. Spencer

**Affiliations:** The Jenner Institute, Nuffield Department of Medicine, University of Oxford, Oxford, UK

**Keywords:** Vaccines, Adaptive immunity, Immunology, Microbiology

## Abstract

Traditional chemical adjuvants remain a practical means of enhancing the immunogenicity of vaccines. Nevertheless, it is recognized that increasing the immunogenicity of viral vectors is challenging. Recently, STING ligands have been shown to enhance the efficacy of different vaccine platforms, but their affectivity on viral-vectored vaccination has not been fully assessed. In this study we used a multi-pronged approach to shed light on the immunological properties and potential mechanisms of action of this type of adjuvant and focused our study on replication-deficient human adenovirus serotype 5 (AdHu5). When the STING ligand 2′3′-cGAMP was mixed with AdHu5, the adjuvant enhanced anti-vector immune responses while decreasing the transgene-specific CD8^+^ T cell response. Studies employing STING-knockout mice and a 2′3′-cGAMP inactive analogue confirmed the aforementioned effects were STING dependent. In vitro assays demonstrated 2′3′-cGAMP induced the production of IFN-β which in turn negatively affected AdHu5 transgene expression and CD8^+^ T cell immunogenicity. In an effort to overcome the negative impact of early 2′3′-cGAMP signaling on AdHu5 transgene immunogenicity, we generated a bicistronic vector encoding the 2′3′-cGAMP together with a model antigen. Intracellular production of 2′3′-cGAMP after AdHu5 infection was able to enhance transgene-specific CD8^+^ T cell immunogenicity, although not to a level that would warrant progression of this adjuvant to clinical assessment. This work highlights the importance of timing of 2′3′-cGAMP administration when assessing its adjuvant capacity with different vaccine modalities.

## Introduction

Four recombinant replication-incompetent adenoviral vaccines were developed and licensed for COVID-19 within 14 months of the discovery of the coronavirus genome sequence^1–4^. They show important single and two dose efficacy, likely related to both antibody and T cell induction, and over a billion doses of these vaccines have been distributed. However, improvements to immunogenicity of the encoded transgene could further enhance the utility of this vaccine platform. Here we focus on one potential approach to enhancing CD8^+^ T cell immunogenicity induced following vaccination with Adenoviral vectors.

In the last 10 years, there has been an increasing body of work investigating the use of stimulator of interferon genes (STING) ligands as potential vaccine adjuvants. STING ligands, such as 2′3′-cGAMP and 3′3′-cGAMP, have been shown to increase humoral and cellular adaptive immune responses when coadministered with protein-based vaccines^5–11^ and inactivated viruses^12,13^. More recently, 2′3′-cGAMP has been shown to enhance CD8^+^ T cell responses in the cancer immunotherapy field^8,14–16^. The low toxicity of 2′3′-cGAMP, due to its relatively low level induction of proinflammatory responses, encourages the potential use of this type of adjuvant across different vaccine platforms^8,12^.

However, the mechanisms of STING action are complex. After ligand binding, STING induces the activation of multiple immune pathways: TBK1/IRF3-induced type I IFN expression, classical inflammation driven by NF_k_B and STAT-6-dependent gene expression^17–20^, with exogenous 2′3′-cGAMP linked to the activation of dendritic cells and therefore antigen processing and presentation^5,7,14,15^, which could promote cross-priming and activation of antigen-specific CD8^+^ T cells^8,14,15^. Even though STING ligands have been extensively studied as chemical adjuvants, to our knowledge, their capacity to enhance viral-vectored vaccine induced responses has yet to be determined. Viral vectors, in particular recombinant adenoviruses, have become of increasing interest due to their widespread use as COVID-19 vaccines and known capacity to enhance both humoral and cell-mediated immune responses^21^. While previous attempts to enhance viral vector immunogenicity through co-administration with traditional emulsion based chemical adjuvants has been largely unsuccessful, most likely due to the innate stimulatory capacity of the viral vectors^22,23^, enhancing the signalling pathways induced by these vectors may prove a more successful approach.

During a viral infection, DNA is sensed by cGAS (cyclic GMP-AMP synthase) resulting in the synthesis of 2′3′-cGAMP which in turn activates STING leading to the production of IRF3 and IFN-β^24,25^. We hypothesized that by increasing the level of 2′3′-cGAMP at the time of viral vector administration could enhance vaccine induced immunity. 2′3′-cGAMP is relatively safe, rapidly hydrolyzed and metabolized in humans and does not cause significant systemic inflammation^5^. In addition, 2′3′-cGAMP has been found to not provoke acute local inflammatory responses, or toxicity in the liver and kidney^9,12^, therefore may be less reactogenic than traditional licensed adjuvants.

In this study we explored the potential of 2′3′-cGAMP to adjuvant viral vector induced immunity. Our results indicate timing of 2′3′-cGAMP administration is critical, as early administration prior to transgene expression reduced immunity due to the increased induction of type I IFNs. However, these negative effects could be overcome by co-expression of 2′3′-cGAMP together with the transgene resulting in enhanced STING activation in later stages of viral infection. As the kinetics of antigen expression will differ between vaccine modalities, this work demonstrates the importance of timing of adjuvant expression, which could have implications for the use of other chemical adjuvants.

## Results

### STING ligand 2′3′-cGAMP has opposing effects on the immunogenicity of AdHu5

**Figure 1:**
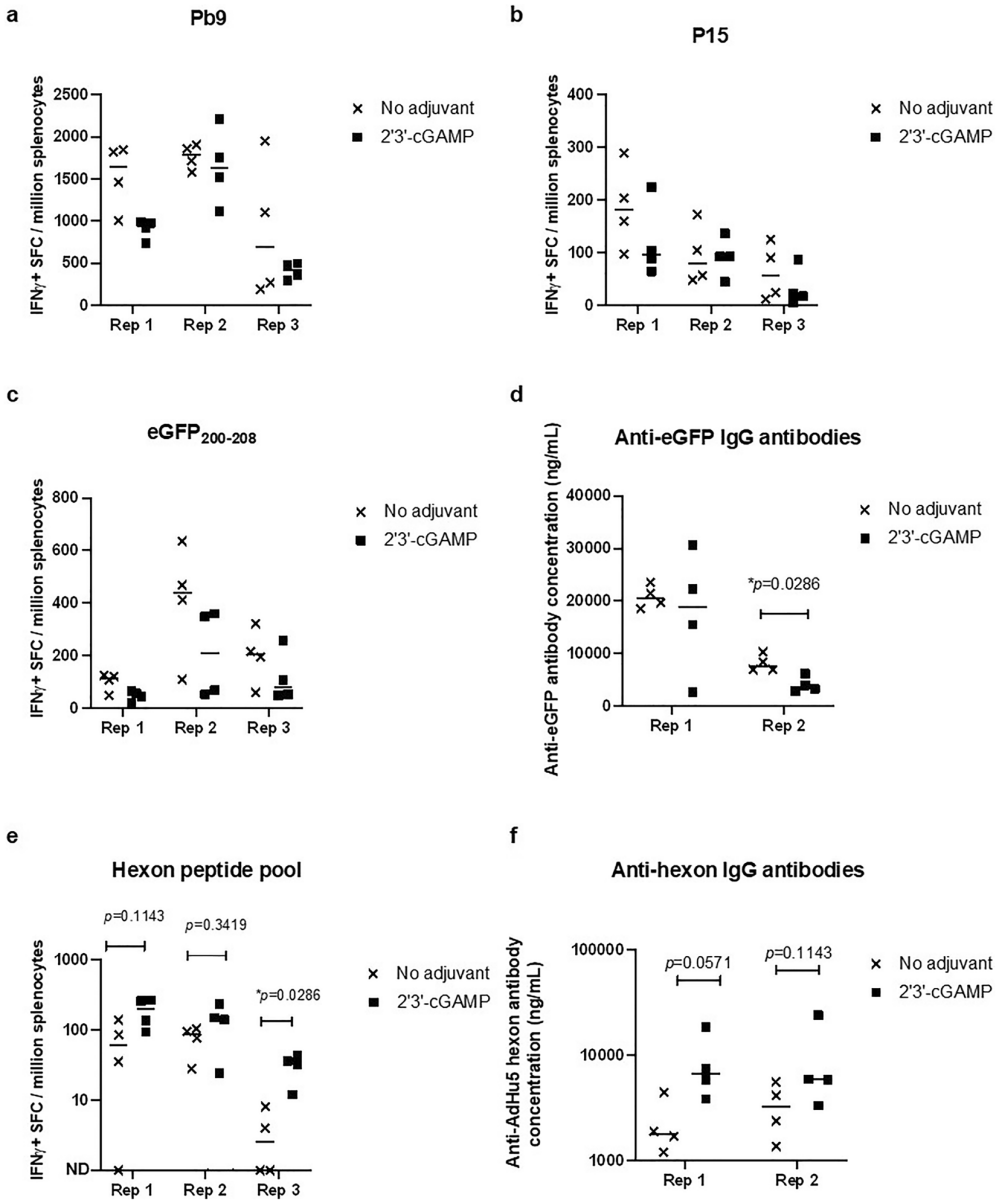
2′3′-cGAMP has opposing effects on AdHu5. Mice (*n* = 4 per experiment) were injected intramuscularly with 10 μg of 2′3′-cGAMP mixed together with 10^7^ IU of AdHu5. T and B cell immunogenicity against encoded antigens and viral capsid proteins were measured 14 days after vaccination. Splenocytes were re-stimulated with Pb9 (**a**), P15 (**b**), eGFP_200-208_ (**c**) and a T cell peptide pool spanning the AdHu5 hexon (**e**) and IFN-γ production measured by ELISpot. For antibody responses, serum was analyzed using IgG ELISA coating the plates with eGFP (**d**) or AdHu5 hexon protein (**f**). For each panel, Rep 1, Rep 2 and Rep 3 represent independent experiments. Black horizontal lines for each mouse group represent the group medians. Statistical analysis: Mann–Whitney test between treatments within a single experiment (no adjuvant versus 2′3’-cGAMP) (*p < 0.05). The sample sizes correspond to the initial intention of gathering evidence of the phenomena*.*

To determine whether activating the STING pathway could enhance vaccine induced immunity, 2′3′-cGAMP was mixed with a replication-deficient AdHu5 that expresses CD4^+^ and CD8^+^ T cell epitopes^26–28^, together with Green Fluorescence Protein (eGFP) to measure induction of antibodies as well as an additional CD8^+^ T-cell immunodominant epitope (Supplementary Figure S1). At 2-weeks post vaccination, a consistent decrease in IFN-γ ELISpot CD8^+^ T cells responses, Pb9 (from a malaria parasite) and eGFP_200-208_ was observed (Fig. 1a–d) in 2′3′-cGAMP vaccinated animals compared to non-adjuvant controls animals (two-way ANOVA main effect analysis: *p = 0.0320 and *p = 0.0484, respectively). A reduced response to the CD4^+^ T cell epitope P15 was also observed, albeit not statistically significant (*p* > 0.05).

The negative impact of 2′3′-cGAMP on the transgene-specific immunogenicity was also observed at different doses of 2′3′-cGAMP and when mice were vaccinated intradermally (Supplementary Figure S2). However, when 2′3’-cGAMP was administered 3 days after viral vector administration, a smaller reduction in immunogenicity was observed, suggesting an impact of time on 2′3′-cGAMP administration (Supplementary Figure S3).

Immunological responses to transgene antigens require virus infection, protein production, antigen processing and presentation, thereby creating a time gap/delay between the presence of injected 2′3′-cGAMP and presentation of vaccine antigens when adjuvant is mixed with the viral vector. To determine whether the detrimental effect was due to timing of antigen presentation, we measured the immune response to early vector proteins, i.e. viral capsid proteins. Mixing 2′3′-cGAMP led to an increase in T and B cell immunogenicity against the hexon proteins (Fig. 1e–f) in individual experiments, although small in each experiment, this was consistent across replicates (two-way ANOVA). This enhancement of early expressed proteins was also found when a virus-like particle R21 malaria vaccine^29^, that does not rely on transgene expression for induction of immunogenicity, was administered together with 2′3-cGAMP (Supplementary Figure S4).

### The adjuvant effects of 2′3′-cGAMP on early AdHu5 antigens is dependent on STING

**Figure 2:**
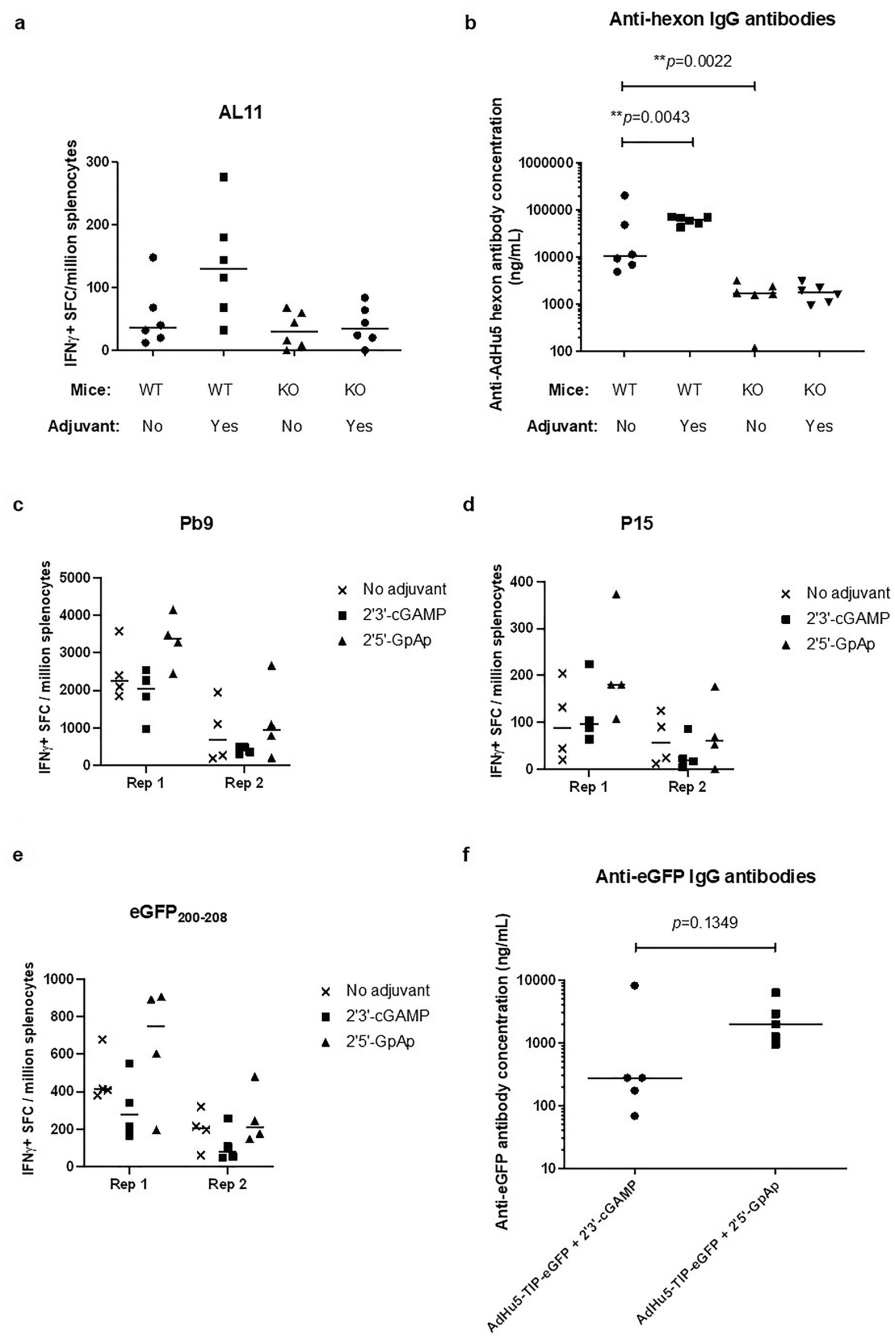
The adjuvant effects of 2′3′-cGAMP on AdHu5 is dependent on STING. STING-knockout and C57BL/6 mice (*n* = 6 per experiment) (panels **a** & **b**) and wild type BALB/c mice (n = 4 per experiment) (panels **c**-**f**) were injected intramuscularly with ~ 10 μg (14.8 nmol) per mouse of 2′3’-cGAMP or 2′5’-GpAp mixed with 10^7^ IU of AdHu5 per mouse. T and B cell responses were measured 14 days after vaccination. Splenocytes were re-stimulated with a T cell peptide pool spanning the AdHu5 hexon protein (**a**), Pb9 (**c**), P15 (**d**) and eGFP_200-208_ (**e**). Splenocyte responses were analyzed using IFN-γ ELISpot. For B cell responses, serum was analyzed using IgG ELISA coating the plates with AdHu5 hexon protein (**b**) or eGFP (**f**). For each panel, Rep 1 and Rep 2 represent independent repetition experiments. Black horizontal lines for each mouse group represent the group medians. Statistical analysis: Mann–Whitney test between treatments within a single experiment (no adjuvant versus 2′3′-cGAMP or 2′5′-GpAp, or knockout mice versus wild type mice) (**p < 0.001).

2′3′-cGAMP interacts with STING, a protein in the endoplasmic reticulum, which leads to subsequent activation of other proteins (IKK, TBK1) and transcription of type I interferon IFN-β and other cytokines and chemokines^30^. To confirm addition of 2′3’-cGAMP was enhancing the response to early AdHu5 proteins in a STING dependent manner, STING-knockout mice^31^ were vaccinated and immune response analyzed. No enhancement of AdHu5-hexon responses were observed in STING KO mice, indicating 2′3-cGAMP mediated enhancement is STING signaling dependent (Fig. 2a and b). 2′3′-cGAMP mediated enhancement of anti-vector IgG responses was also shown to be STING dependent, as an increase in anti-GFP titers was observed in wild type C57BL/6 mice but not in STING KO mice (Fig. 2b), while no difference in the level of transgene mediated immunogenicity (AL11 SIV-gag CD8^+^ T cell epitope) was observed between STING KO and wildtype mice (Supplementary Figure S5).

We also compared the immunological effects of 2′3′-cGAMP to its linear (non-functional) analogue (2′5′-GpAp)^32^ in wild type BALB/c mice. The results indicated that 2′3′-cGAMP induced lower Pb9 and eGFP_200-208_*-*specific immune responses compared to its linear analogue (two-way ANOVA main effect analysis: **p = 0.0095 and *p = 0.0402, respectively) (Fig. 2c–f). The effect against the peptide P15 remained non-significant (*p* = 0.1195), providing further evidence the detrimental effect on the Pb9 and eGFP_200-208_ (both CD8^+^ T cell peptides) was due to STING activation and not to physicochemical property of the adjuvant.

### IFN-β induced by exogenous 2′3′-cGAMP correlates with AdHu5 CD8^+^ T cell transgene immunogenicity

AdHu5 transgene expression is a major determinant of the transgene-specific CD8^+^ T cell immunogenicity^33^, with adjuvants that induce the production of type I IFNs capable of reducing AdHu5 transgene expression and thus transgene-specific immunogenicity. Having observed activation of STING leading to a negative impact on transgene-specific CD8^+^ T cell immunogenicity (Pb9 and eGFP_200-208_), we sought to determine if a similar type 1 IFN mediated mechanism was leading to the observed effects of mixing 2′3’-cGAMP, which is known to induce IFN-β^24,34^.

**Figure 3:**
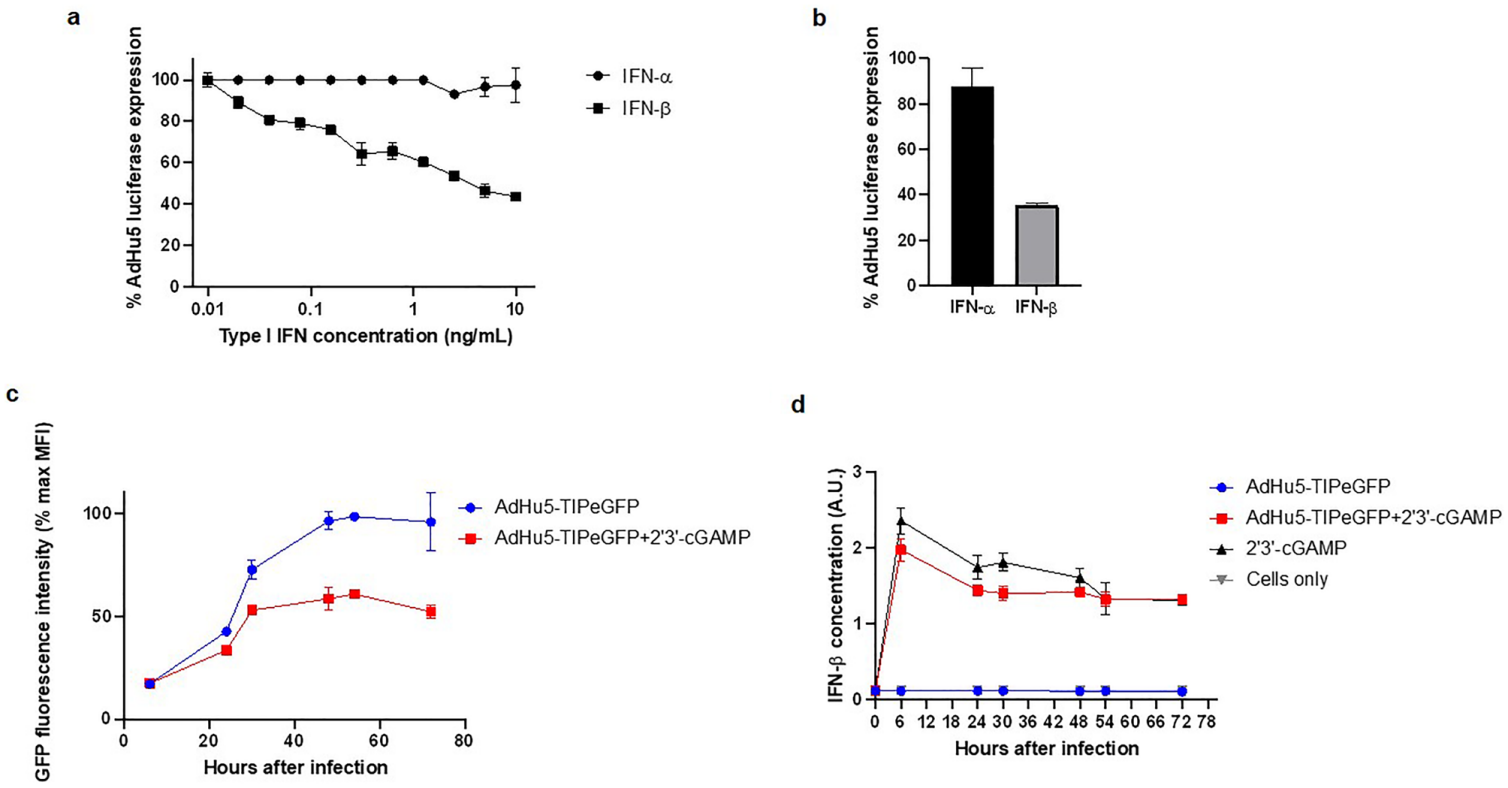
IFN-β induced by exogenous 2′3’-cGAMP correlates with AdHu5 transgene expression. (**a**) Percentage of AdHu5 luciferase expression was dependent on the concentration of human IFN-α/β (MOI 12). (**b**) AdHu5 luciferase expression at a concentration of 5 ng/mL of murine IFN-α/β in the murine cell line Hepa1-6 (MOI 12). Luciferase activity was measured 48 h after viral infection. (**c**) Effect of 2′3′-cGAMP (83.3 mM) on the kinetics of eGFP expression from AdHu5-infected Hepa1-6 cells (MOI 1000). (**d**) Kinetics of IFN-β production from the same experiment as (**c**). For panels **a** & **b**, IFN-α is used as a negative control ^38^. For all experiments, mean values + SD are shown for each time point and IFN concentration; these values were obtained from 3 technical replicates.

To determine if exogenous IFN-β could directly diminish AdHu5 transgene expression, a cell-based assay using AdHu5 expressing a luciferase gene was employed. When IFN-β was mixed with AdHu5 early during in vitro infection, the luciferase expression of AdHu5 was significantly diminished (Fig. 3a and b). Following eGFP production over the course of AdHu5 infection showed that the addition of 2′3-cGAMP lead to overall decrease in eGFP expression detected as early as 24 h post-infection (Fig. 3c). Addition of 2′3-cGAMP, IFN- β was detected in the cell culture supernatant as early as 6 h post-infection, but AdHu5 infection alone did not lead to IFN-β production (Fig. 3d). Importantly addition of non-functional 2′5-GpAp did not lead to reduction in transgene expression (Supplementary Figure S6). Overall, the results confirmed that 2′3’-cGAMP induced the production of IFN-β (Fig. 3 c and d) and simultaneously reduced AdHu5 transgene expression.

### Optimization of the delivery of 2′3′-cGAMP to enhance transgene-specific CD8^+^ T cell responses

Having identified early STING signaling negatively impacting transgene immunogenicity, yet wanting to optimize the use of 2′3′-cGAMP to enhance CD8^+^ T cell responses, we generated a bicistronic vector to ensure simultaneous transgene and STING signaling.

**Figure 4:**
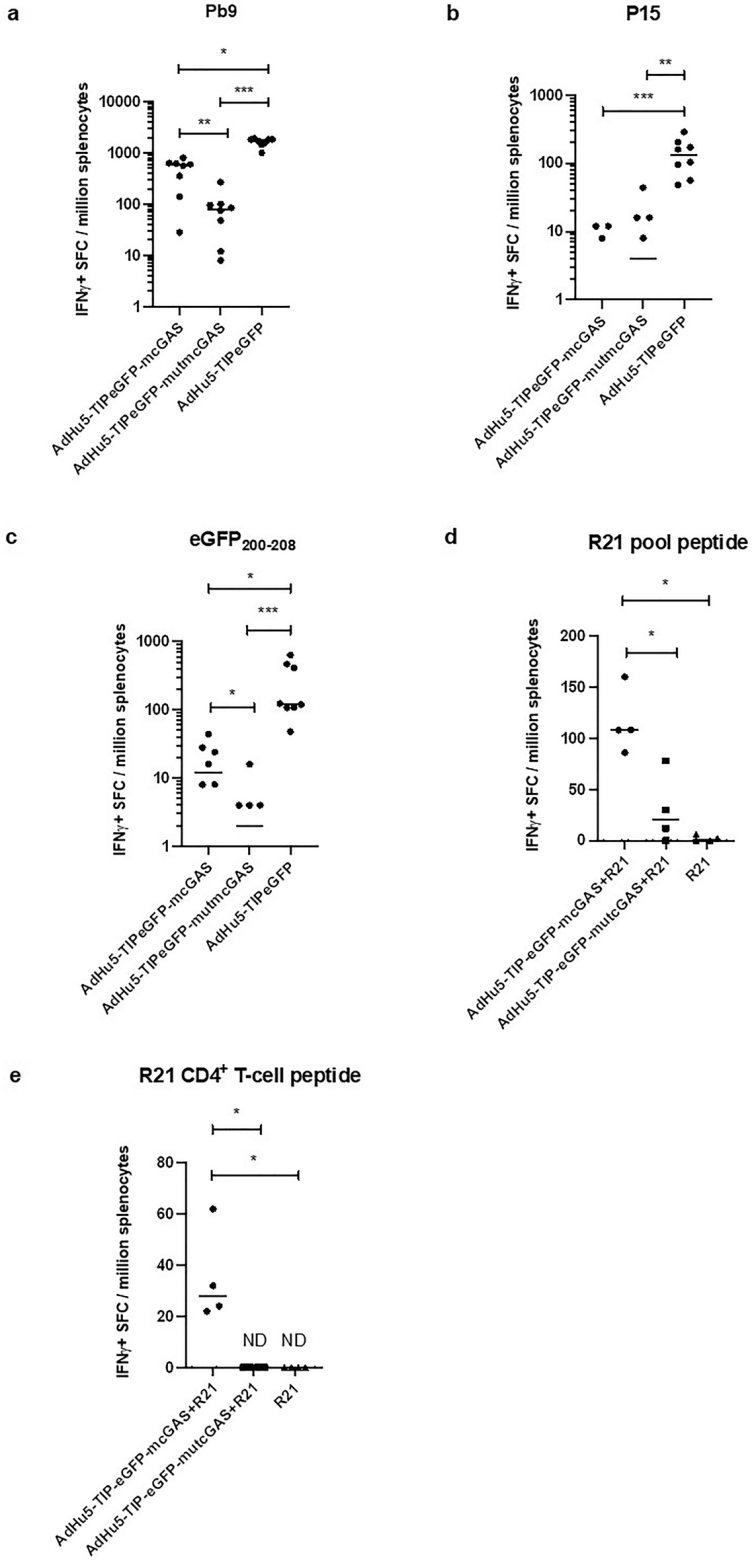
Immunological effects of the optimized delivery of 2′3′-cGAMP. BALB/c mice (*n* = 4–8 per experiment) were injected intramuscularly with 10^7^ IU per mouse of AdHu5 co-expressing cGAS and transgene antigens (AdHu5eGFP-mcGAS). For panels **d** & **e**, AdHu5eGFP-mcGAS was mixed with 0.5 μg of R21 per mouse. Negative control was the AdHu5 co-expressing a mutated inactive version of cGAS (AdHu5eGFP-**mut**mcGAS). Immune responses were measured 14 days after vaccination using IFN-γ ELISpot. Splenocytes were re-stimulated with Pb9 (**a**), P15 (**b**), eGFP_200-208_ (**c**), a T cell peptide spanning the CSP of R21 (**d**) and a CD4^+^ T cell peptide from the CSP of R21 (**e**). Horizontal lines in each panel represent the group medians. Statistical analysis: Kruskal–Wallis test with Dunn’s post hoc, *p < 0.05, **p < 0.01, ***p < 0.001. For comparison of cGAS-expressing AdHu5s: Mann–Whitney test: *p < 0.05, **p < 0.01.

We constructed an AdHu5 co-expressing the transgene antigens (Pb9, P15, eGFP_200-208,_ AL11 and eGFP) and the natural 2′3′-cGAMP synthase, also known as cGAS^24^ (Supplementary Fig. S1). As a control, the same antigen insert was co-expressed with inactive version of cGAS containing relevant inactivating point mutations^24^. The expression and activity of the encoded cGAS was confirmed in vitro (Supplementary Fig. S7). In vivo immunogenicity showed that the delivery of 2′3′-cGAMP after viral infection (by means of co-expressing it as a transgene) enhanced the CD8^+^ T cell immunogenicity against Pb9 and eGFP_200-208_ (Mann–Whitney test: **p = 0.0070 and *p = 0.0467, respectively), but not the CD4^+^ T cell immunogenicity against P15 (*p* > 0.05) (Fig. 4a–c). Enhancement of the CD8 + T cell response was also observed when AdHu5 vectors were administered by intradermal injection (*p = 0.0286 for Pb9 and *p* = 0.400 for eGFP_200-208_) and same trend observed at different viral vector doses (*p* = 0.3719 and *p = 0.0286 at doses 10^6^ and 10^8^, respectively) (Supplementary Figure S8 & S9). However, the transgene immunogenicity induced by cGAS-expressing AdHu5 was lower than control AdHu5 expressing only the transgene antigens (Fig. 4a–c), suggesting an impact of the co-expression system. Reduced but equivalent level of eGFP was observed with both cGAS-expressing vectors compared to the AdHu5 (Supplementary Figure S7 a), but only cGAS, and not the mutant, induced IFNβ production in vitro. Since the only difference between vaccines was their ability to deliver 2′3′-cGAMP, we consider the above experiments still valid for assessing the effect of 2′3′-cGAMP on the transgene-specific CD8^+^ T cell immunogenicity co-expressed from the same vector (as opposed to previous mixing experiments).

We then aimed to expand the potential applicability of the delivery of 2′3′-cGAMP after viral vector infection. We mixed the cGAS-expressing AdHu5s with R21, a vaccine previously tested and found to be compatible with 2′3′-cGAMP adjuvantation (Supplementary Figure S4). The mouse experiments showed that intracellular delivery of 2′3’-cGAMP after viral vector infection also enhanced the T cell immunogenicity of R21 as assessed by ELISpot assays (Fig. 4d, e).

Overall, these results indicate that the intracellular delivery of 2′3′-cGAMP after viral vector infection could serve to enhance the transgene-specific CD8^+^ T cell immunogenicity of AdHu5, while also enhancing the immunogenicity of other vaccines being mixed in the vaccine formulation.

## Discussion

Previous work has studied the use of STING ligands for adjuvanting traditional protein based vaccines^5–13^, to our knowledge, the utility of STING ligands as adjuvants for viral-vectored vaccination has not been fully explored. In this study we assessed and optimized the use of 2′3’-cGAMP for its ability to enhance viral-vectored induced responses.

We demonstrated that exogenous 2′3’-cGAMP has opposing immunological effects on AdHu5 antigens, dependent on the time of protein expression. When mixed with the viral vector, the response to early antigens, e.g. hexon proteins, were enhanced but transgene-specific CD8^+^ T cell responses were reduced. These results agree with a previous reports where the in vivo synthesis of c-di-GMP, another STING ligand, by AdHu5 was shown to enhance anti-vector T cell immunogenicity, while at the same time diminishing the transgene-specific CD8^+^ T responses of another AdHu5 mixed in the vaccine formulation^35^, but the mechanisms of action of the c-di-GMP were not assessed.

The data presented in this study also indicates the immunological effects of the adjuvant 2′3′-cGAMP is dependent on STING signaling. This agrees with a previous study establishing the influence of the cGAS/STING pathway on the anti-vector humoral responses of AdHu5^36^. In this study no influence of cGAS/STING pathway on transgene expression was reported. Adenoviral DNA is sensed by the cGAS/STING pathway inducing production of type I IFNs^37^, but in the case of AdHu5, these type I IFNs are believed to be present at a level too low to have a substantial impact on transgene expression^37^. Therefore, it is possible that studies concerning cGAS/STING knockout mice fail to detect a difference on the AdHu5 transgene immunogenicity, simply because type I IFN levels are too low to have an impact. The situation differs when adding an adjuvant that enhances type I IFNs production through the same pathway, as with 2′3′-cGAMP. In this case, the induction of type I IFNs levels high enough to cause an effect on AdHu5 transgene expression. This is also exemplified by the finding that poly I:C mixed with AdHu5 triggers the induction of type I IFNs affecting transgene expression and CD8^+^ T cell immunogenicity, in a STING-dependent manner^37^.

The capacity of IFN-β to directly block AdHu5 transgene expression has not, to our knowledge, been previously described, but is supported indirectly by studies in IFNAR-deficient mice (mice lacking the receptor for the type I IFNs α & β) where the detrimental effect of adjuvant-induced type I IFNs is eliminated^37^. Since IFN-α appears not to directly affect AdHu5 transgene expression^38^, it seems more likely that IFN-β is mediating this effect. In addition, human IFN-β has been described to have a detrimental effect on the replication of different adenoviral serotypes in vitro^39^.

In this study we showed that intracellular delivery of 2′3′-cGAMP, by means of a bicistronic vaccine, was able to enhance transgene-specific CD8^+^ T cell immunogenicity. Replication-deficient AdHu5 has a characteristic steep peak of transgene expression at day 1 after vaccination^40,41^, in contrast, the production of cytokines and chemokines induced by 2′3′-cGAMP injected by the same route of vaccination has a peak of expression of 6 h after injection, with levels returning to baseline 24 h after 2′3′-cGAMP injection^42^. We hypothesize that by inducing STING activation after AdHu5 infection it abrogates the early antiviral response induced by the addition of 2′3′-cGAMP, enabling more transgene expression and enhancing immunogenicity.

The enhancement of protein-based vaccines immunogenicity observed with 2′3′-cGAMP, is strongly linked to the activation of dendritic cells^5,14^, which enhances dendritic cell activation and vaccine antigen processing and presentation in a STING-dependent manner. This enhanced T cell immunogenicity is likely to be a product of increased MHC Class II antigen presentation and cross-priming by dendritic cells and macrophages that subsequently will induce the activation of both CD4^+^ and CD8^+^ T cell responses. Indeed 2′3′-cGAMP has been recently found to facilitate the cross-priming of antigen-specific CD8^+^ T cell responses when mixed in the vaccine formulation^8^. Therefore, it can be hypothesized that the same mechanism might be playing a role in the enhancement of CD8^+^ T cell responses of viral vectors.

Unfortunately, the translation applicability of the cGAS-expressing AdHu5 (AdHu5-TIP-eGFP-mcGAS) is limited as immunogenicity was considerably lower than the AdHu5 only expressing the antigens, suggesting issue with vaccine design or stability. Nevertheless, having shown induction of type 1 IFN can affect Adenovirus infection, blocking the effects of type I IFNs may be a future area to explore be used to enhance the immunogenicity of this viral vector.

The work described in this study sought to determine whether activation of the STING pathway could enhance immune response induced following viral vectored vaccination. While 2′3-cGAMP was able to enhance early expressed antigens, over production of type 1 IFN led to reduction in transgene antigen expression, therefore had a negative impact on vaccine induced responses, highlighting the need to assess candidate adjuvants across multiple vaccine modalities prior to clinical translation.

## Material and methods

### Viral vectors and antigens

AdHu5-TIPeGFP is a replication-deficient (E1/E3-deleted) viral vector expressing the antigen TIPeGFP under the promoter human EF-1α^43^. The TIPeGFP is a synthetic antigen comprising the peptides Pb9, P15 and AL11. These peptides are fused to an enhanced Green Fluorescence Protein (eGFP) (Fig. S1). The *Plasmodium berghei* CD8^+^ T cell epitope Pb9 is an H-2K^d^-restricted immunodominant epitope from the circumsporozoite protein^44^. The peptide P15 is a CD4^+^ T cell peptide from *Mycobacterium tuberculosis* Antigen 85A^44,45^. The AL11 peptide is an H-2D^b^-restricted CD8^+^ T cell peptide from the gag protein of the Simian Immunodeficiency Virus (SIV)^46^. The inclusion of the eGFP enables the study of antibody responses against an encoded antigen and the study of an additional CD8^+^ T cell peptide named eGFP_200-208_^47^.

Replication-deficient (E1/E3-deleted) AdHu5-mcGAS-TIPeGFP and AdHu5-mutmcGAS-TIPeGFP were constructed as previously described^43^. The mouse cGAS gene (Genbank accession number: KC294567.1) was purchased from InvivoGen (France). The cGAS gene was then cloed into a shuttle vector by In-Fusion cloning (Clontech). The cGAS gene was inserted immediately downstream the human cytomegalovirus immediate early (CMV-IE) promoter in the shuttle vector. This vector already contained a TIPeGFP synthetic gene under the control of the human Elongation Factor promoter EF1-α.

The catalytically inactive version of the cGAS gene (E211A and D213A, described in^24^) was created by site-directed mutagenesis (PCR) of the aforementioned shuttle vector already containing the cGAS and TIPeGFP genes. These two created shuttle vectors contained AdHu5-specific E1 recombination sites flanking the two expression cassettes (cGAS and TIPeGFP) for posterior homologous recombination with a BAC parental genome of AdHu5.

Gateway® homologous recombination between the shuttle vectors containing the mouse cGAS and TIPeGFP antigen and the AdHu5 BAC parental genome was performed. This homologous recombination technique is described in^43^. During the homologous recombination, the biscistronic cassette was inserted in the E1 locus of the AdHu5 parental genome. Resulting AdHu5 genomes were then derived in HEK293A cell lines (Invitrogen, Cat. R705-07). The resultant viruses were purified by CsCl gradient ultracentrifugation, as previously described^48^. The virus titers were determined on HEK293A cells using anti-hexon immunostaining assay using the QuickTiter™ Adenovirus Titer Immunoassay kit (Cell Biolabs Inc).

The virus-like particle R21 is expressed in yeast using the small Hepatitis B surface antigen (HBsAg) fused to the C-terminus region of part of the Circumsporozoite protein (CSP) of *Plasmodium falciparum*^29^.

### Statement on mouse ethics—project license

Mice were used in accordance with the UK Animals (Scientific Procedures) Act under project license number P9804B4F1 granted by the UK Home Office. The experimental protocols were approved by the University of Oxford Animal Care and Ethical Review Committee. Animals were group housed in individually ventilated cages under specific pathogen free conditions, with constant temperature, humidity and with a 12:12 light–dark cycle (8 am to 8 pm). For induction of short-term anaesthesia, animals were anaesthetized using vaporized IsoFlo®. All animals were humanely sacrificed at the end of each experiment by an approved Schedule 1 method. All efforts were made to minimize suffering. The study is reported in accordance with ARRIVE guidelines.

### Mouse immunogenicity

Female BALB/c.OlaHsd (Envigo, U.K.), C57BL/6 J.OlaHsd (Envigo, U.K.) purchased in batches from suppliers for each experiment and sub-divided into groups on arrival in the facility. **B6(Cg)-Sting1**^***tm1.2Camb***^**/J** (STING KO mice) bred in house (^31^) with mice from each litter mixed across groups to ensure spread of ages and sexes. Mice injected intramuscularly or intradermally with different doses of AdHu5 (10^6^–10^8^ IU per mouse). R21 was administered at dose of 0.5 μg. For all AdHu5 experiments, immune responses were measured 14 days after vaccination. For R21-only experiments, immune responses were measured 3 weeks after boosting (3 weeks between prime and boosting vaccinations). 1–30 μg of 2′3′-cGAMP and 2′5′-GpAp per mouse were used either mixed together with the vaccine preparation or administered as a separate injection as indicated in the figure legend.

### Serum IgG ELISA

For the analysis of serum IgG antibody responses against viral vectors and virus-like particles, 96-well Nunc-Immuno Maxisorp ELISA plates were coated with 50 μL of enhanced Green Fluorescent Protein (eGFP) (1 μg/mL, Millipore), NANP_6_ polypeptide (2 μg/mL, ProImmune) or AdHu5 hexon protein (1 µg/mL, BioRad) in PBS.

A standard endpoint ELISA protocol was followed as previously described^49^. Serum was diluted 10 or 100-fold in PBS-Tween (PBS/T). This initial dilution was further diluted across the 96-well ELISA plate in 50 μL volumes. For each mouse sample, the ELISA test was performed in duplicates. Plate was incubated for 1–2 h before a washing step. Subsequently, goat anti-mouse total IgG conjugated to alkaline phosphatase (Sigma) and pNPP (*p*-nitrophenylphosphate) tablet substrate (SIGMA) were used in the assay. OD405 signal from each well was read using an ELISA microplate reader. The endpoint titers were obtained after normalization of the ELISA OD405 values against the positive control and expressed in ng/mL. Serum from a naïve BALB/c mouse was used as a negative control.

### Ex-vivo IFN-γ ELISpot

Splenocytes were harvested for analysis with ex-vivo IFN-γ ELISpot, described in^50,51^. In brief, single cell suspensions of splenocytes were treated with ammonium chloride-potassium (ACK) lysis buffer and cells plated on IPVH-membrane plates (Millipore) coated with 5 µg/ml anti-mouse IFN-γ (AN18) (Mabtech). Cells were re-stimulated with Pb9 (1 μg/mL, ProImmune), eGFP_200-208_ (1 μg/mL, ProImmune), P15 (2 μg/mL, ProImmune), AL11 (1 µg/mL, ProImmune), AdHu5 hexon T cell peptide pool (PepTivator) (1 µg/mL, Miltenyi Biotech), T cell peptide pool spanning the C-terminus region of the circumsporozoite protein of *P. falciparum* (1 µg/mL, ProImmune) or CD4^+^ T cell peptide from the C-terminus region of the *P. falciparum* CSP (CSP_326-345_)^52^ (1 µg/mL, Mimotopes Pty Ltd).

Plates were incubated for 18–20 h and IFN-γ spot forming cells (SFC) were detected after staining with 1 mg/mL of anti-mouse IFN-γ biotin (Cat No. R46A2, Mabtech), followed by 1 mg/mL of streptavidin–Alkaline Phosphatase (Mabtech). Development was performed using an alkaline phosphatase (AP) conjugate substrate kit (BioRad, U.K.).

IFN-γ spots were counted using an ELISPOT Reader System ELR04 (AID GmbH). Samples were analyzed in technical duplicates. The final data is presented as spot-forming cells (SFC) per million splenocytes, after subtracting the background (number of IFN-γ SFC in wells containing cells and media only).

### In vitro adjuvant effects

For the analysis of the effect of 2′3′-cGAMP and IFN-β on the AdHu5 transgene immunogenicity by fluorescence microscopy, Hepa1-6 cells were plated in a 96-well black plate (clear bottom) and incubated overnight. Replication-deficient AdHu5 expressing eGFP (AdHu5-TIPeGFP) was used to infect the cells at a MOI of 1000. 2′3′-cGAMP at a concentration of 83.3 mM was added to each well, and the plate was incubated at 37 °C for 1–2 days. The mean fluorescence intensity (MFI) from each well was determined at different time points after infection. The MFI was determined by using a fluorescence microscope (Leica DMI 3000 B) and the software Q-Capture Pro 7 (Teledyne Qimaging). The images obtained were analyzed using ImageJ software (NIH). The MFI was normalized against the maximum MFI recorded during the experiment.

For the analysis of luciferase activity, Huh7.5 (ATCC CRL-2117) and Hepa 1–6 cells (ATCC CRL-1830) were plated in a white 96-well plate and incubated overnight. Different concentrations of human IFN-β and IFN-α (1–10 ng/mL; R&D Systems) were added across the plate and then a single concentration of AdHu5 expressing the firefly luciferase gene was added (MOI 12). The replication-deficient AdHu5 expressed the firefly luciferase gene under a human CMV promoter (virus described in^53^). For analyzing the effect of mouse IFN-α and β, a single concentration of 5 ng/mL was used. Luciferase activity was read 48 h after infection using Bright-Glo Luciferase Assay System (Promega) as per the manufacturer´s instructions. The plates were read using Thermo Scientific Varioskan® Flash and the software SkanIT™ (ThermoFisher Scientific).

### Statistical analysis

The Mann–Whitney test, the Kruskal–Wallis test with Dunn’s post hoc and the two-way ANOVA main effect analysis were used for analyzing immunological responses. A parametric t-test was used for the pairwise comparison of the *in-vitro* experiment. GraphPad Prism was used for all the statistical analyses and to plot the data.

## Supplementary Information

Supplementary Information.

## Data Availability

The datasets generated during and/or analysed during the current study are available from the corresponding author on reasonable request. The data are not publicly available due to privacy concerns.
