# Supplementary material for: STING-pathway modulation to enhance the immunogenicity of adenoviral-vectored vaccines
Source: Sci Rep. 2022 Aug 24;12:14464. doi: 10.1038/s41598-022-18750-3 (PMC9401198; doi:10.1038/s41598-022-18750-3)
Supplement: Supplementary file 1 — Supplementary Information. [file 41598_2022_18750_MOESM1_ESM.docx]

SUPLEMENTAL INFORMATION

**STING-pathway modulation to enhance the immunogenicity of adenoviral-vectored vaccines**

Eriko Padron-Regalado*, Marta Ulaszewska, Alexander D. Douglas, Adrian V.S. Hill & Alexandra J. Spencer*.

FIGURES


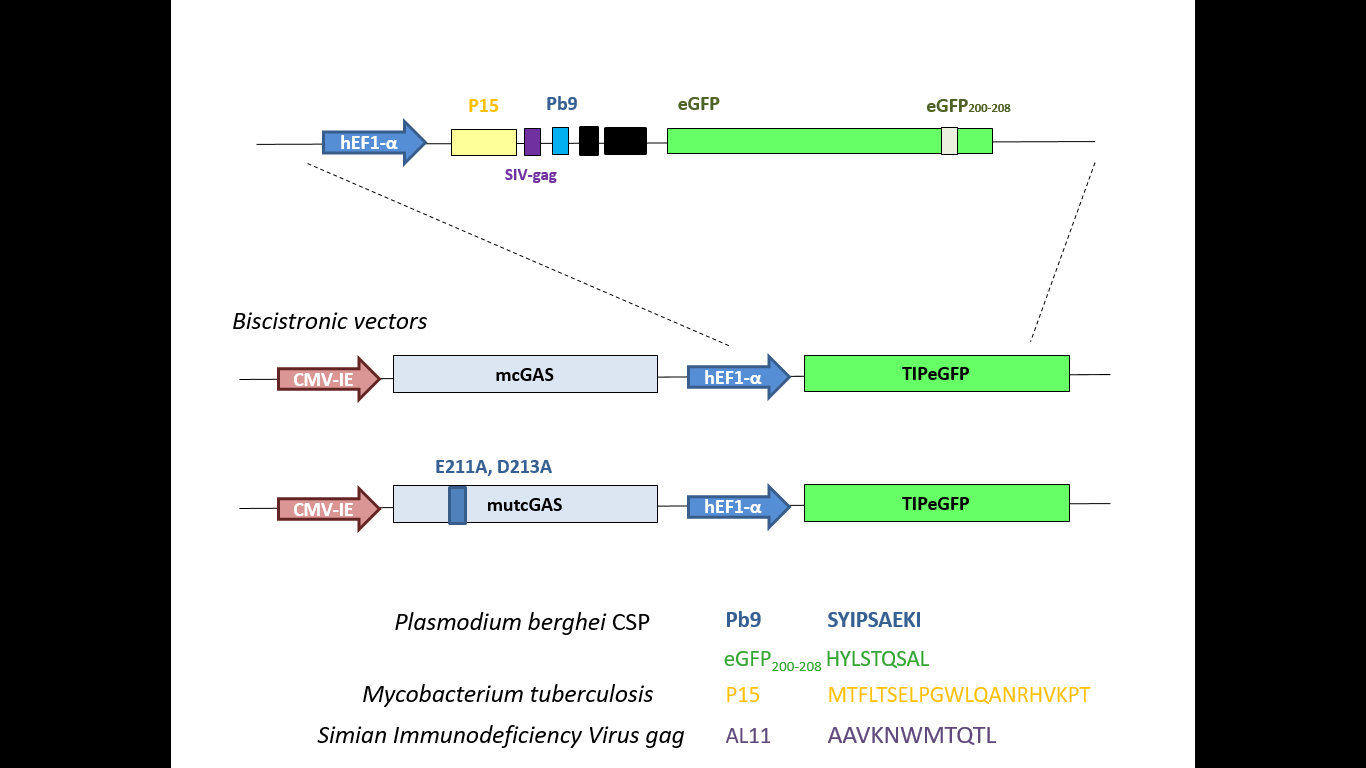


**Supplementary Figure 1. Scheme of the transgene peptides encoded by the viral vectors.** The synthetic polypeptide comprises the CD4^+^ T cell peptide P15 fused to the CD8^+^ T cell peptides SIV-gag (AL11) and Pb9. These peptides are fused to the N-terminus to the enhanced Green Fluorescence Protein (eGFP) that bears an additional CD8^+^ T cell peptide denominated eGFP_200-208_. The complete synthetic polypeptide has been denominated TIPeGFP (acronym of Tuberculosis, Influenza, Plasmodium, enhanced Green Fluorescence Protein). Black boxes correspond to the peptides PyCD4 and Py3 epitopes which were not measured in the present study. For the biscistronic vectors, the mcGAS gene was inserted upstream the hEF1-α-TIPeGFP cassette. mcGAS gene expression was driven by the promoter CMV-IE. The mutated version of the biscistronic vector differed in the inclusion of two inactive mutations in the mcGAS gene (E211A and D213A).


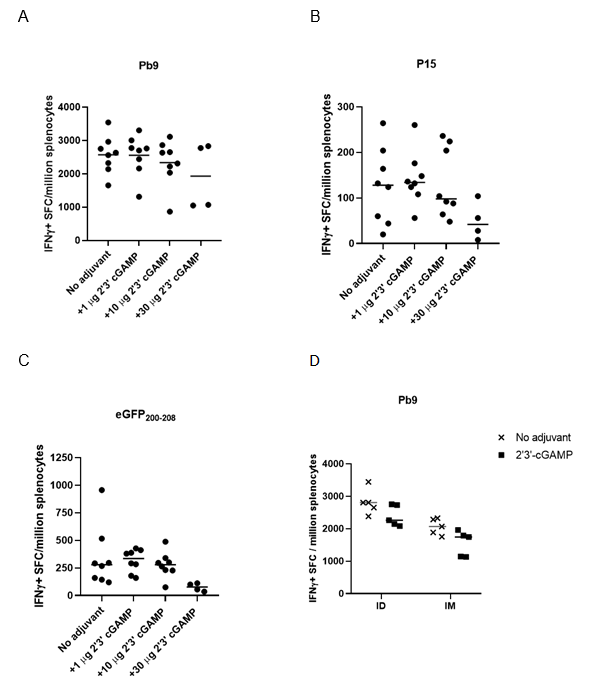


**Supplementary Figure 2.** **Effect of 2’3’-cGAMP on the transgene immunogenicity of AdHu5.** The effect on the transgene immunogenicity of AdHu5 of increasing doses of 2’3’-cGAMP (1-30 µg per mouse) were evaluated using a spleen *ex vivo* IFN-γ ELISpot after stimulation with peptides Pb9 (A), P15 (B) and eGFP_200-208_ (C). The AdHu5 was employed in a dose of 10^7^ IU per BALB/c mouse. For panel D, effect of the adjuvant (10 µg per mouse) on the frequency of IFNγ^+^ CD8^+^ T cell responses against Pb9 (ID=intradermal vaccination, IM=intramuscular vaccination). For all panels, horizontal lines represent the medians per group with each mouse displayed as a single point. Statistical analysis: Kruskal-Wallis test with Dunn’s post hoc, *p*>0.05. Mann-Whitney test (*p*>0.05).


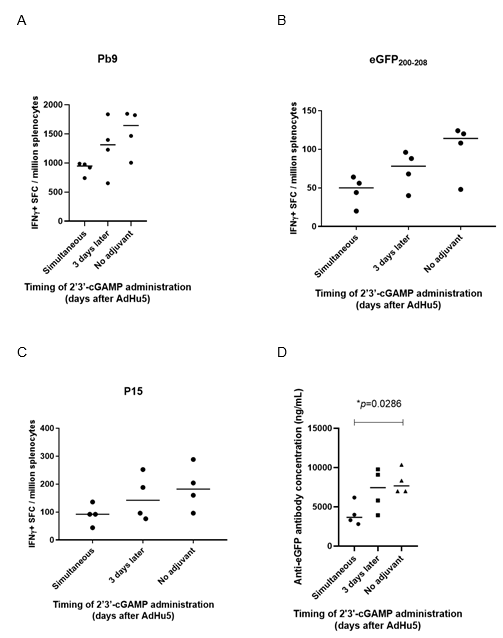


**Supplementary Figure 3. Effect of the time of administration of 2’3’-cGAMP on the AdHu5 transgene immunogenicity.** A dose of AdHu5 expressing the antigen TIP-eGFP was administered intramuscularly (10^7^ IU per BALB/c mouse) and the adjuvant 2’3’-cGAMP (10 μg per mouse) was co-administered (“Simultaneous”) or injected at the same thigh region 3 days after viral vector vaccination (“3 days later”). The immunogenicity of the viral vector injected alone is shown for comparison (“No adjuvant”). Spleen IFNγ^+^ T cell responses against the transgene peptides Pb9 (A), eGFP_200-208_ (B) and P15 (C) were evaluated at day 14 after vaccination. D. Serum IgG anti-eGFP antibody responses at the same time point as A-C. Statistical analysis: Mann-Whitney test, *p<0.05. Time points represent the median values and range (n=4).


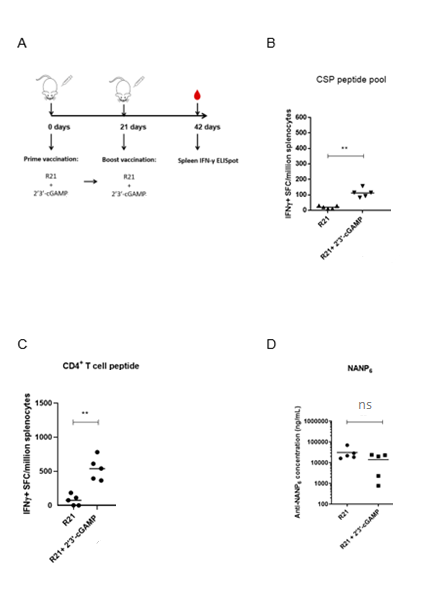


**Supplementary Figure 4.** **Effect of 2’3’-cGAMP on the immunogenicity of R21**. A. Scheme showing the vaccination regimens with R21 and adjuvants 2’3’-cGAMP used in this study. R21 vaccine formulations (with or without 2’3’-cGAMP) were injected intramuscularly to BALB/c mice on day 0 and boosted with the same vaccine formulation 3 weeks after. Immunological responses were measured 3 weeks after boosting. Vaccine doses used: R21 (0.5 μg/mouse) and 2’3’-cGAMP (10 μg/mouse). B. IFNγ^+^ T cell responses from spleens against the C-terminus of the CSP of the R21 vaccine (CSP T cell peptide pool). C. IFNγ^+^ CD4^+^ T cell response from spleens using a CD4^+^ T cell peptide from the C terminus of the CSP. D. Endpoint IgG serum ELISA titers against the NANP_6_ polypeptide of the C terminus of the CSP. Statistical analysis: Mann-Whitney test (**p<0.01). Horizontal lines represent means of mouse groups.

| 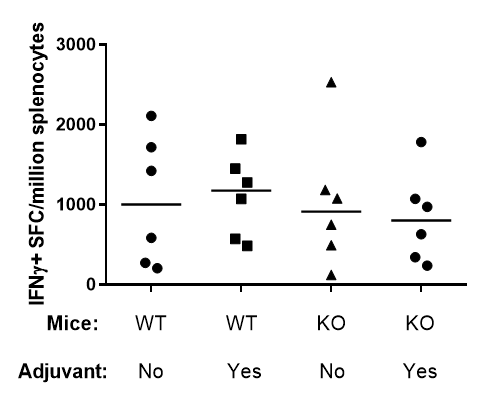 |
| --- |

**Supplementary Figure 5. Effect of the adjuvant 2’3’-cGAMP on the AdHu5 transgene immunogenicity in wild type and STING-knockout mice.** IFNγ^+^ CD8^+^ T cell responses against the AdHu5-encoded peptide AL11 and determined by an ex-vivo spleen ELISpot. Mice were vaccinated with 10^7^ IU AdHu5 per mouse with or without 2’3’-cGAMP (10 μg per mouse). Spleen samples were analyzed at the peak of the immune response (day 14 after vaccination). Statistical analysis: Mann-Whitney test, p>0.05. The horizontal lines represent the medians for each mouse group.

| 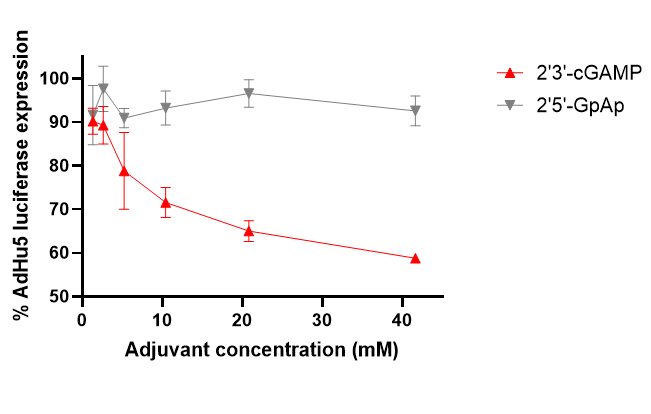 |
| --- |

**Supplementary Figure 6. Effect of 2’3’-cGAMP on the AdHu5 transgene expression.** Hepa 1-6 cells were plated in a 96-well plate and exposed to serial concentrations of 2’3’-cGAMP 24 hours after AdHu5 infection (MOI 12). Luciferase activity was measured 48 hours after viral infection. 2’5’-GpAp was used as a negative control. Data points correspond to mean of three technical replicates + SD.


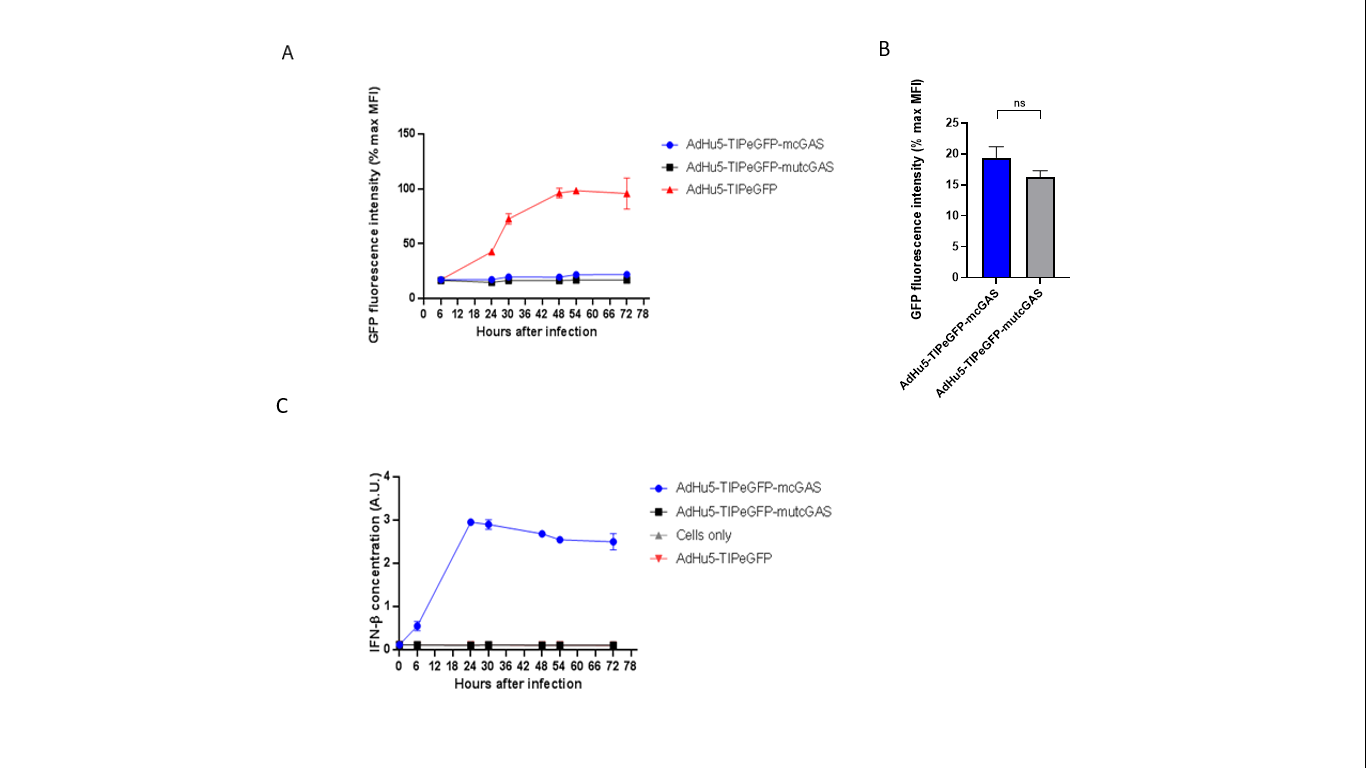


**Supplementary Figure 7.** **Evaluation of cGAS and TIP-eGFP expression from the biscistronic AdHu5s.** A. eGFP expression kinetics after infection of Hepa 1-6 cells with the AdHu5s (MOI 1000). B. Comparison of the eGFP expression between the bicistronic vectors 48 hours after infection. C. IFN-β expression kinetics as determined by an anti-mouse IFN-β ELISA at the same experimental conditions as A. Data points from A and B correspond to the mean values of 3 technical replicates + SD. For B, statistical analysis was performed using the unpaired Student´s t-test; ns= non-significant.


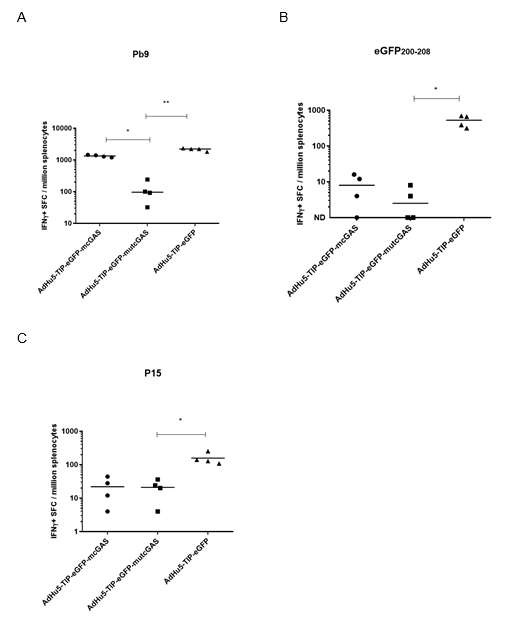


**Supplementary Figure 8. Effect on the AdHu5 transgene immunogenicity of the expression of cGAS by the intradermal route of vaccination.** A. IFNγ^+^ CD8^+^ T cell responses against the peptide Pb9 (A) and eGFP_200-208_ (B) and IFNγ^+^ CD4^+^ T cell responses against the peptide P15 (C) as measured by spleen ELISpot at day 14 after vaccination (10^7^ IU per BALB/c mouse). Statistical analysis: Kruskal-Wallis test with Dunn’s post hoc, *p<0.05, **p<0.01, n=4. For difference in immunogenicity between the biscistronic vectors: Mann-Whitney test, *p<0.05. Horizontal lines represent the group medians for each mouse group.


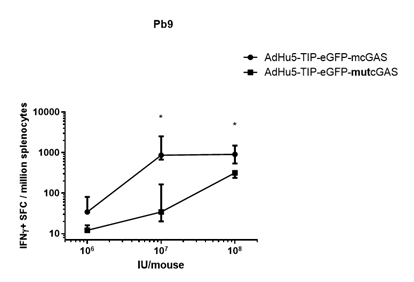


**Supplementary Figure 9. Effect of the cGAS/STING pathway activation on the AdHu5 transgene immunogenicity at different doses of the viral vectors.** BALB/c mice were vaccinated intramuscularly and Pb9-specific IFNγ^+^ CD8^+^ T cell responses were measured 14 days after vaccination by spleen ELISpot. Statistical analysis: Mann-Whitney test for each viral vector dose, *p<0.05, n=4. Data-points represent the median values + range.
